# Conformational Studies of Glucose Transporter 1 (GLUT1) as an Anticancer Drug Target

**DOI:** 10.3390/molecules24112159

**Published:** 2019-06-07

**Authors:** Suliman Almahmoud, Xiaofang Wang, Jonathan L. Vennerstrom, Haizhen A. Zhong

**Affiliations:** 1 Department of Pharmaceutical Sciences, College of Pharmacy, University of Nebraska Medical Center, Omaha, NE 68198, USA; suliman.almahmoud@unmc.edu (S.A.); xiaofangwang@unmc.edu (X.W.); jvenners@unmc.edu (J.L.V.); 2 Department of Chemistry, University of Nebraska at Omaha, Omaha, NE 68182, USA

**Keywords:** glucose transporter 1 (GLUT1), conformation, docking, anticancer, drug design

## Abstract

Glucose transporter 1 (GLUT1) is a facilitative glucose transporter overexpressed in various types of tumors; thus, it has been considered as an important target for cancer therapy. GLUT1 works through conformational switching from an outward-open (OOP) to an inward-open (IOP) conformation passing through an occluded conformation. It is critical to determine which conformation is preferred by bound ligands because the success of structure-based drug design depends on the appropriate starting conformation of the target protein. To find out the most favorable GLUT 1 conformation for ligand binding, we ran systemic molecular docking studies for different conformations of GLUT1 using known GLUT1 inhibitors. Our data revealed that the IOP is the preferred conformation and that residues Phe291, Phe379, Glu380, Trp388, and Trp412 may play critical roles in ligand binding to GLUT1. Our data suggests that conformational differences in these five amino acids in the different conformers of GLUT1 may be used to design ligands that inhibit GLUT1.

## 1. Introduction

Glucose transporter 1 (GLUT1) is a membrane protein encoded by the solute carrier family 2A1 (SLC2A1) genes [1]. GLUT1 is a member of sugar transporter subfamily of the major facilitator superfamily (MFS) [2]. GLUT1 has a conserved core fold that consists of 12 transmembrane helices folded into two distinguishing domains, the amino (N) and carboxyl (C) terminal domains [3]. Each domain has six sequential transmembrane helixes (TMs) that are folded into a pair of ‘3+3′ inverted repeats [3]. TM7 and TM10 are broken segments, hence termed TM7a/7b and TM10a/10b, respectively [4]. Four short intracellular α-helices (IC1–4) connect the N-terminal and C-terminal domains [4]. The C domain provides the main substrate-binding site for glucose [5]. GLUT1 transports monosaccharides including d-glucose and d-galactose, but does not transport fructose [6,7]. GLUT1 is expressed in diverse tissues with distinct kinetic behavior and different substrate affinity [8].

Cancer cells transport more glucose than normal cells due to their rapid growth and high rate of aerobic glycolysis, a phenomenon called the Warburg effect [9,10,11]. GLUT1 is upregulated in many cancers such as brain [12], breast [13], lung [14], kidney [15], ovary [16], prostate [17], and colon [18]. In addition, stimulation of oncogenes like such as KRAS [14], BRAF [19], c-myc [20], and p53 [21], and transcription factors such as hypoxia-inducible factor-1a (HIF-1) [22] upregulate GLUT1 expression. GLUT1 inhibition results in reduction of cancer-cell proliferation and apoptosis [23,24]. While GLUT1 is overexpressed in many tumors, we note that in the brain, glucose transport is facilitated by both GLUT1 and GLUT3 [25,26,27,28]. However, GLUT3 has greater affinity for glucose and higher capacity than GLUT1 in the brain (Km for d-glucose of 3.4 mM for GLUT1, and Km for 2-deoxy-glucose of 5 and 1.4 mM for GLUT1 and GLUT3, respectively) [26,27]. A ligand selectively bound to GLUT1 rather than GLU3 would be able to spare the GLUT3 inhibition and, thus, would minimize the potential neurotoxicity due to GLUT inhibition. Several small molecule GLUT1 inhibitors and chemotypes have been described including resveratrol [29], naringenin [30], phloretin [31], cytochalasin B [32], WZB117 [33], STF-31 [34], pyrazolopyrimidines [35], phenylalanine amides [32], and (1H-pyrazol-4-yl)quinoline [36]. 

GLUT1 transports substrates by the alternating access mechanism, which involves the “rocker-switch” movement and the “gated pore” mechanisms [37,38]. GLUT1 changes from an outward-open (OOP) conformation, which opens to the extracellular to take up glucose, to an inward-open (IOP) conformation, which allows the release of glucose to the intracellular cytoplasm via intermediate outward-occluded (OOC) and partially inward-occluded (PIO) conformations [39,40,41,42]. Substrate-free GLUT1 favors the OOP conformation [5]. Once the substrate binds to the C domain of the GLUT1, the transporter shifts to the IOP conformation to release glucose [5]. 

The only crystal structures of human GLUT1 are for the IOP conformation [5,32]. Since only the IOP conformation is available for the human GLUT1 protein, we built homology models for other conformations using GLUT1 homologous crystal structures that have already adopted the needed conformations. For example, crystal structures of human glucose transporter 3 (GLUT3), which has an 86% sequence similarity to GLUT1, were obtained for the OOC and OOP conformations [4]. The crystal structure of *Escherichia coli* proton: Xylose symporter (XylE), which has a 63% sequence similarity to GLUT1, were obtained for the PIO and inward-occluded (IOC) conformations [43,44]. The essential amino acids interacting with glucose are conserved between Xyle and GLUT1 [43].

It is critical to determine which conformation is preferred by bound ligands because the success of structure-based drug design depends on the appropriate starting conformation of the target protein. To identify the most favorable conformation for GLUT1 inhibitor binding, and to determine important amino residues that may be responsible for ligand interactions, we ran a series of docking studies of reported GLUT1 inhibitors against GLUT1 in different conformations: Outward-open (the OOP), partially outward occluded (POO), outward occluded (OOC), inward-open (IOP), and partially inward occluded (PIO) conformations. The docking scores and the enrichment factor (EF) as well as the ligand protein interactions suggested that the GLUT1 prefers the IOP conformation for ligand binding. 

## 2. Results and Discussion

### 2.1. Homology Modeling of GLUT1

The only crystal structures described for GLUT1 (PDB ID: 4PYP, 5EQG, 5EQH, and 5EQI) are for the IOP [5,32]. The OOP, OOC, POO, and PIO conformations for GLUT1 have not yet been identified by X-ray crystallographic structures; hence, we constructed these models through homology modeling. The amino acid residue alignment of GLUT1 with GLUT3 and XylE proteins showed that they have largely conserved glucose-binding residues and the highly conserved residues between these three proteins are highlighted in yellow (Figure S1 in the Supplementary Materials). GLUT1 has 66% sequence identity and 80% similarity with GLUT3; GLUT1 has 29% sequence identity and 49% similarity with XylE [43]. 

**Figure 1:**
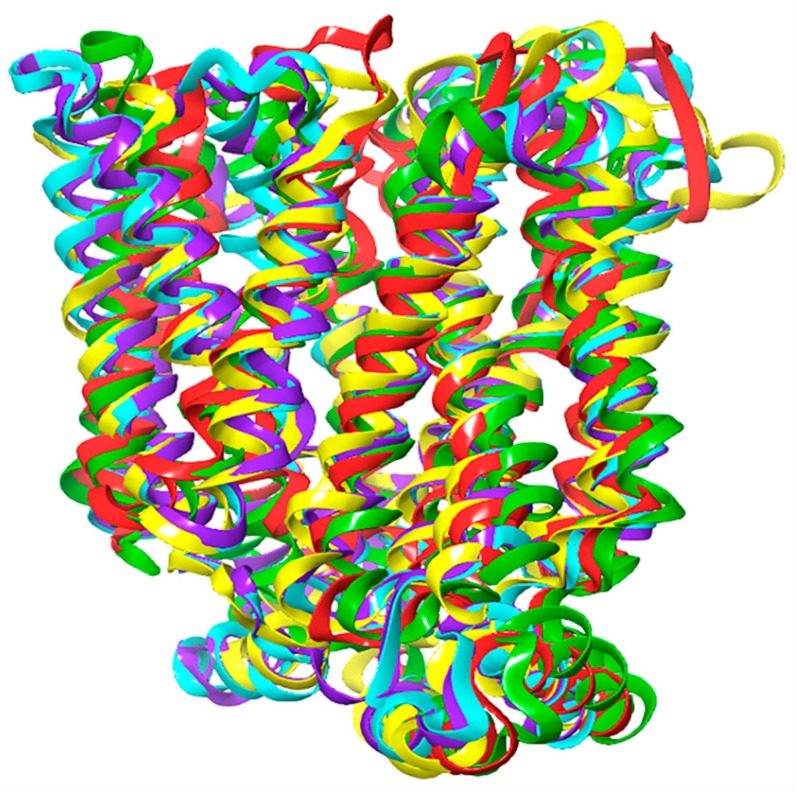
An overview of working model of GLUT1: The function of GLUT1 depends on conformational change. The inward-open (IOP) conformation, green, was adopted from PDB ID: 5EQG; the outward-open (OOP) conformation, cyan, was constructed by homology modeling of PDB ID: 4ZWC; the partially outward occluded (POO) conformation, yellow, was constructed by homology modeling of PDB ID: 4GBZ; the outward-occluded (OOC) conformation, violet, was constructed by homology modeling of PDB ID: 4ZW9; the partially inward occluded (PIO) conformation, red, was constructed by homology modeling of PDB ID: 4JA3.

The OOP and OOC were built through homology modeling by using the crystal structures of human GLUT3 (PDB ID: 4ZWC, and 4ZW9) [4] as templates. Bacterial XylE, a GLUT1 homology model (PDB: 4GBZ) was used to model the POO conformation [43], and the template (PDB: 4JA3) was used to build the PIO conformation [44] (Figure 1). Structural alignment of GLUT1 to different homolog models shows that most of the secondary structures are conserved between these models and that the orientation of the folds differs, resulting in the OOP, POO, OOC, PIO, and IOP conformations (Figure 1).

Homology modeling is one of the most successful methods to build and predict the tertiary structure of a protein that has not been defined [45]. Homology modeling depends on sequence alignment of proteins. If the sequences of two proteins are similar, they will have comparable tertiary structure folding [45]. Amino acid residue alignment of GLUT1 with GLUT3 and XylE proteins exhibited that they have notable conserved residues in the sequences, especially at the glucose binding site residues (Figure S1 in the Supplementary Materials). GLUT1 has high sequence identity (66%) and similarity (80%) with GLUT3, and GLUT1 has sequence identity (29%) and similarity (49%) with XylE [43]. The accuracy of the models was evaluated by comparing the backbone atoms of the homology modeling and the X-ray template and measuring the root mean-square deviation (RMSD) between the backbone atoms of the homology modeling and the template after superposition. The RMSDs were 0.59, 0.56, 1.27, and 1.49 Å for the conformations OOP, OOC, POO, and PIO, respectively. The low RMSDs (0.55–1.49 Å, less than the threshold of 2 Å) indicates that these homology models are reliable. 

In addition, the backbone structures of the homology models of GLUT1 were evaluated by the Ramachandran’s plots assessment (Figure S2 in the Supplementary Materials). The OOP model had 90%, 10%, and 0.50% of the residues, respectively, assigned as the “most favored”, “additionally allowed”, and “generously allowed” regions. Moreover, no residue was found in the “disallowed” region. The OOC model had 90%, 9%, and 1% in the three “allowed” regions, and only two residues (0.50%) were in the “disallowed” region (Tyr52 and Gln469). The POO model had had 79%, 16%, and 3% in the three “allowed” regions, and five residues (1.3%) were in the “disallowed” region (Val39, Ile128, Gln305, Tyr308, and Ser365), while the PIO had 78%, 18%, and 3% in the three “allowed” regions, and six residues (1.6%) were in the “disallowed” region (Leu115, Ile259, Tyr268, Val307, Tyr308, and Trp363). The residues in the disallowed regions are not involved in the glucose binding site. Thus, both the RMSD values and the Ramachandran’s plots assessment confirm that these homology models of hGLUT1 should be useful for ligand docking.

### 2.2. Docking Scores and Validation

**Figure 2:**
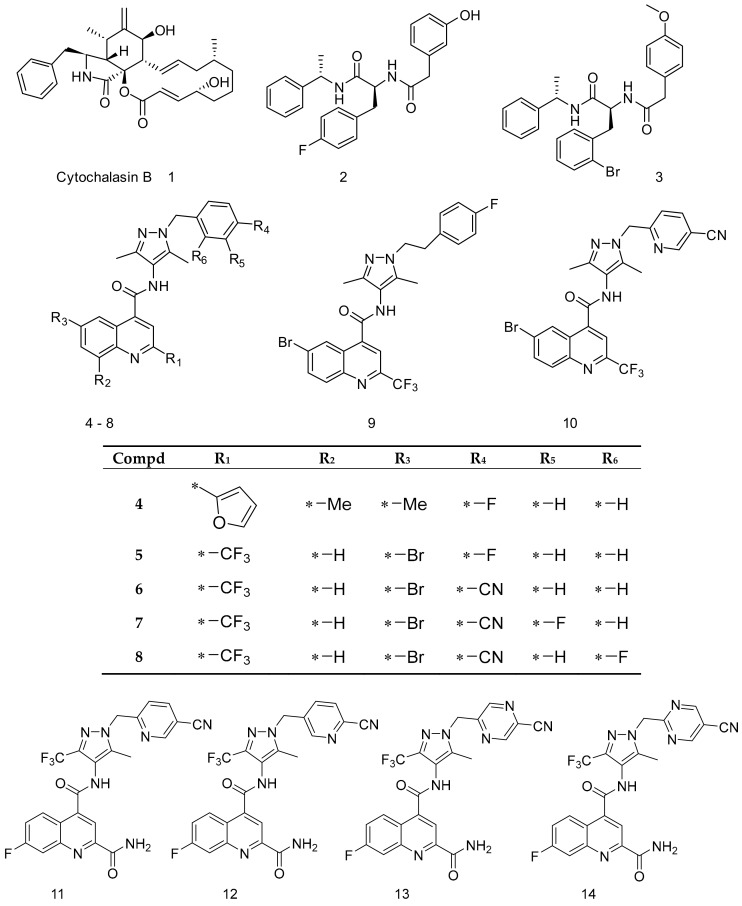
Structures of GLUT1 inhibitors (**1**–**14**) used in docking studies.

**Figure 3:**
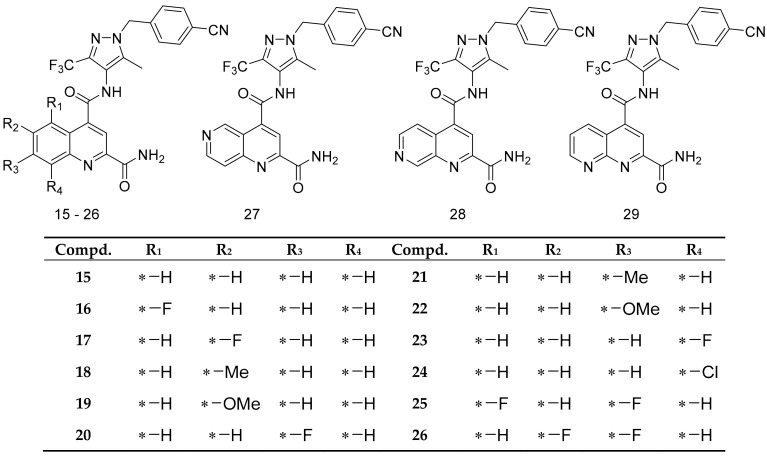
Structures of GLUT1 inhibitors (**15**–**29**) used in docking studies.

**Figure 4:**
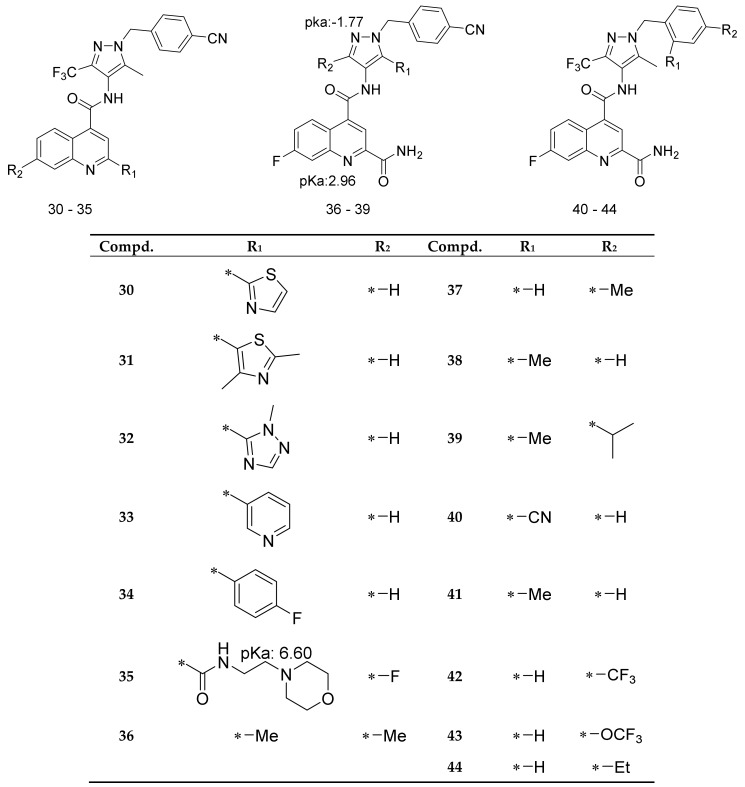
Structures of GLUT1 inhibitors (**30**–**44**) used in docking studies.

To identify amino acids that are essential for ligand binding, we performed docking studies with 44 GLUT1 inhibitors [32,36] (Figure 2, Figure 3 and Figure 4). 

To evaluate the accuracy of the Glide Dock, we first assessed the similarities between the docked poses and original conformations in the crystal structures. The root mean square deviation (RMSD) between the docked poses and the native conformations found in the crystal structures is used to measure the effectiveness of a docking program. A pose with an RMSD < 2.0 Å is considered to be good [46]. The superposition of the Glide generated docked poses of ligands and the native conformation in the crystal structure (Figure S3 in the Supplementary Materials) show that the Glide program accurately identified the native conformation and, thus, can be reliably used to identify the binding conformations of other ligands. The RMSD between two poses for 5RE in 5EQG, 5RF in 5EQH, 5RH in 5EQI, MAL in 4ZWC, BGC in 4GBZ, GLC in 4ZW9, and BGC in 4ZW9 were 1.62, 1.49, 1.09, 0.14, 0.61, 0.94, and 0.06 Å, respectively. These data validate the accuracy of Glide Dock for this study. 

The docking of GLUT1 inhibitors (Figure 2, Figure 3 and Figure 4) to the conformational models of GLUT1 (Table 1) reveal that Glide scores are close to the experimental free energies (ΔGexp) where ΔGexp was approximated as (ΔGexp = RTlnIC_50_) for the IOP conformation. In the inward-open (IOP) conformation, the difference between ΔGexp and Glide scores for the majority of GLUT1 inhibitors was less than 2 kcal/mol. The mean errors (ΔΔG) (Table 2) between ΔGexp and Glide scores (ΔGpred) was −0.97 kcal/mol, which is lower than ±1.00 for the IOP conformation. The predicted docking scores for many compounds were very close to those obtained from the experiments. For instance, the ΔΔG for cytochalasin B was only 0.10 kcal/mol (Table 1). The ΔΔG for the IOP model was listed in Table 1. The ΔΔG for POO, OOP, and PIO were −1.37, −1.44, and −1.14 kcal/mol, respectively. The mean absolute error (MAE), which is the mean absolute value of ΔΔG, was the lowest for the IOP conformation. The MAE for the IOP conformation was the lowest 1.45 kcal/mol, while the MAE for POO, OOP, and PIO was 1.75, 1.82, and 1.68 kcal/mol, respectively. In addition, the root mean square error (RMSE) (Table 2) for the IOP was the lowest at 1.79, whereas RMSE for POO, OOP, and PIO was 2.07, 2.05, and 1.92, respectively. Finally, there were no Glide scores from the OOC. In sum, these data suggest that the IOP is the most favorable conformation for ligand binding. 

In addition to the statistical results between ΔGexp and Glide scores, the enrichment factor (EF) is another very valuable approach to measure the accuracy of a docking program; the higher the EF, the more accurate the docking model [47]. To evaluate the EF values, we obtained 508 drug-like molecules from the NCI database (Table S1 in the Supplementary Materials) and docked these molecules along with GLUT1 inhibitors to all GLUT1 conformations. EF is the ratio between the percentage of active ligands in the particular subset and the percentage in the total database [47]. We obtained the highest EF values for the IOP conformation (Table 3), further confirming that IOP is the preferred conformation for ligand binding since it recognizes the largest number of active inhibitors. We calculated the EFs based on the top 1%, 5%, or 10%; all three cases show that IOP has the best EFs. The OOP conformer and the PIO conformer also show good EF values, suggesting that ligands may be able to bind to these conformations as well. However, the partial outward open (POO) conformation had very low EF values. The EF for best case scenario, which all active molecules are identified in the top percentage poses, is 13.44. The EF of 10.45 in the IOP suggests that this model is the most ideal one among all studied conformations as the EF is the closest to the ideal EF.

### 2.3. Binding Interaction of GLUT1 Inhbitors

**Figure 5:**
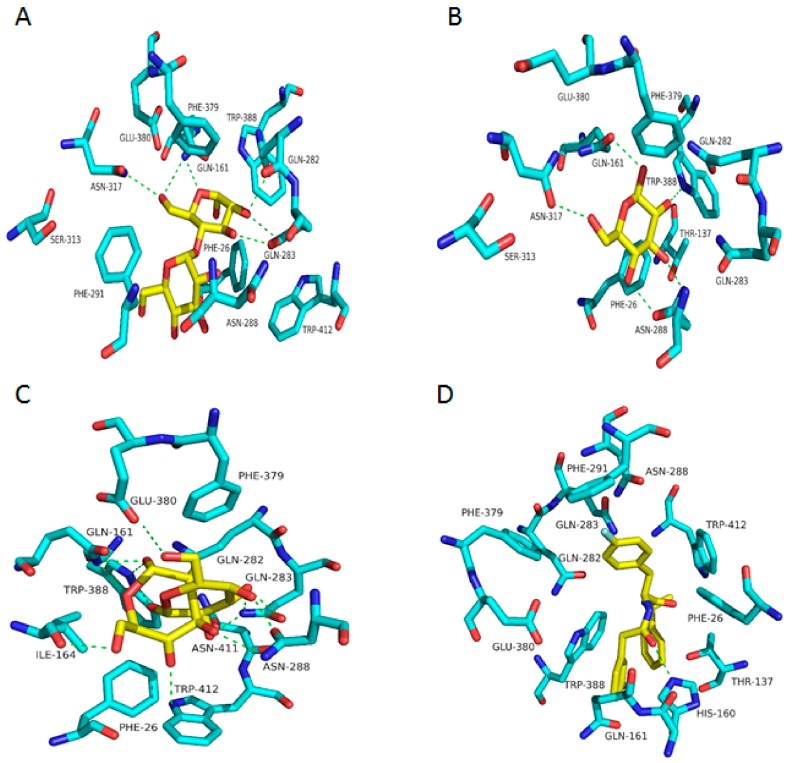
Interactions between crystal ligands and their conformation: The H-bond interactions are shown as green dotted lines. (**A**) Interaction between MAL and GLUT1 in the OOP. (**B**) Interaction between BGC and GLUT1 in the POO. (**C**) Interaction between BGC, GLC, and GLUT1 in the OOC. (**D**) Interaction between 5RE and GLUT1 in the IOP.

As the validity of Glide dock was confirmed, the protein–ligand interactions for the docked poses could then be identified. The homology model of the OOP reveals that one of glucose substructures of maltose forms five H-bonds with four polar residues of GLUT1: Gln161 (2 H-bonds), Gln282, Gln283, and Asn317. In the OOC, β-glucose forms five H-bonds with Gln161, Gln282, Gln283, Asn317, and Trp388. In POO, β-glucose and α-glucose form seven H-bonds with residues: Gln283 (3 H-bonds), Asn288 (2 H-bonds), Asn317, and Trp388. In the IOP conformation, the small molecules interact with Trp388. Our docking studies were in good agreement with the crystal structures of different conformations of GLUT1 (Figure 5). 

**Figure 6:**
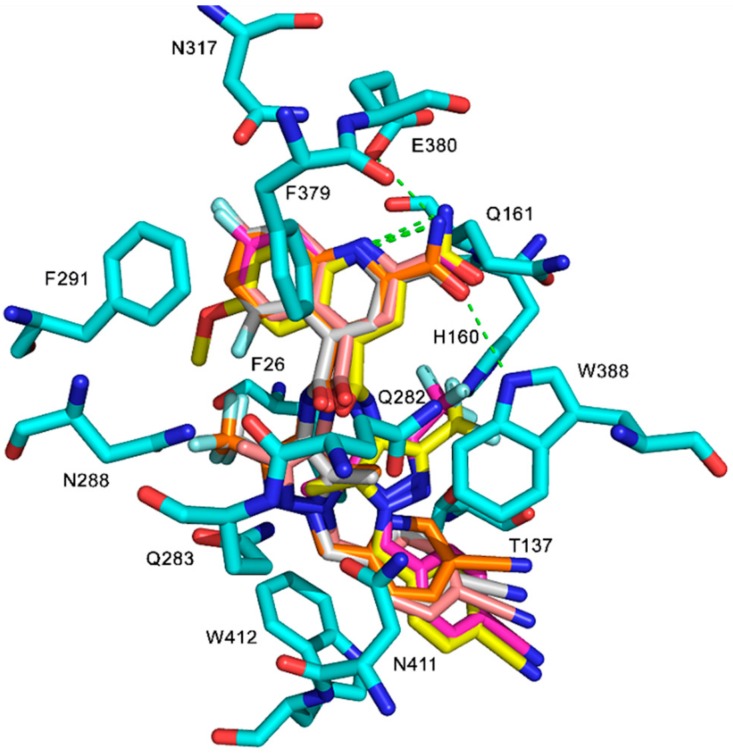
Ligands interactions between 11, 19, 20, 25, and 37 and GLUT1: The H-bond interactions are shown as green dotted lines, and the π–π stacking interaction are shown as chocolate dotted lines. Ligands color code: 11: Carbons, gray; oxygen, red; nitrogen, blue; fluoro, cyan; 19: Carbons, yellow; oxygen, red; nitrogen, blue; fluoro, cyan; 20: Carbons, magenta; oxygen, red; nitrogen, blue; fluoro, cyan; 25: Carbons, orange; oxygen, red; nitrogen, blue; fluoro, cyan; 37: Carbons, tint; oxygen, red; nitrogen, blue; fluoro, cyan. Amino acids residues color codes: Carbons, green; oxygen, red; nitrogen, blue.

**Figure 7:**
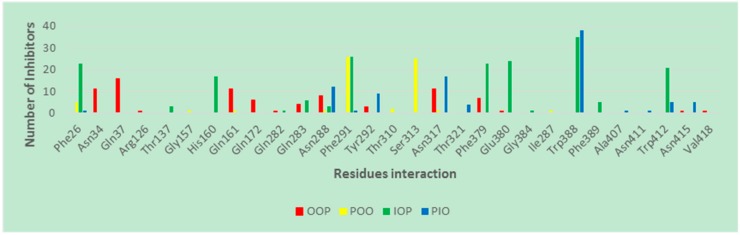
Interacting residues of GLUT1 with inhibitors at different GLUT1 conformations.

Trp388 plays an important role in the access of GLUT1 between the OOP conformation and the IOP conformations [28,48]. Mutation of Trp388 with Leu (W388L) evidently reduced the rate of conformation interchange, consequently decreasing the glucose influx activity [48]. Therefore, an inhibitor that can bind with Trp388 is able to prevent the rotation of Trp388 into the binding site and inhibit glucose uptake. In addition, Trp412 and Phe379 play important roles for the glucose transport function of GLUT1 [49,50]. Therefore, Phe379, Trp388, and Trp412 have important roles in GLUT1 function and glucose uptake. We investigated the interacting residues for ligand binding in different conformations. The protein–ligand interactions between GLUT1 inhibitors and the four GLUT1 models are listed in Table S2 in the Supplementary Materials. Table S2 shows that the majority of GLUT1 inhibitors form H-bonds with Trp388 and π–π stacking via residues Phe379 and Trp412 in the IOP conformation. The inhibitors are able to bind with Phe379, Trp388, and Trp412 once the GLUT1 is in the IOP conformation because these residues are exposed to the IOP binding site (Figure 6). In the OOP and POO conformations, inhibitors do not interact with Trp388 because the indole ring of Trp388 in the OOP is unreachable [4,32]. In the PIO conformation, inhibitors have H-bond interactions with Trp388, but are not able to interact with Phe379 and Trp412. To visualize the important binding residues that interact with ligands, we numerated the number of occurrences that such residues interacted with ligands and presented this frequency in Figure 7. Figure 7 shows that Trp388, Phe379, Glu380, and Trp412 are responsible for ligand-binding to the IOP conformation. Finally, no ligand is well fit to the OOC conformation in the grid generated in Glide. 

In addition, Phe291 and Glu380, which are highly accessible in the binding site in the IOP conformation (Figure 6), may be important residues for ligand interactions, as illustrated in Figure 7. Many GLUT1 inhibitors form H-bonds with Glu380 and π–π stacking interactions with Phe291. Moreover, some ligands have interactions with Phe26 (π–π stacking) and His160 (H-bonds) of GLUT1 (Table S2 in Supplementary Materials and Figure 7), suggesting a possible role for these two residues in GLUT1 binding. These residues may be future targets for site-directed mutagenesis to define their role in GLUT1 function and glucose uptake. In contrast, the number of ligand interactions with these residues is low in the OOP, POO, and PIO conformations.

**Figure 8:**
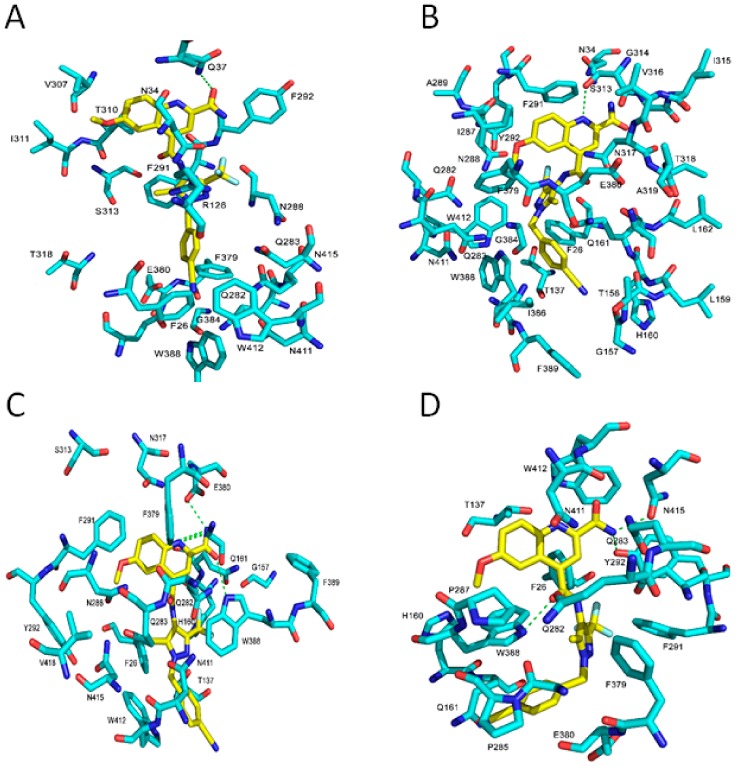
Molecular contact map between 19 and GLUT1 in different conformations: The H-bond interactions are shown as green dotted lines. (**A**) The OOP conformation. (**B**) The POO conformation. (**C**) The IOP conformation. (**D**) The PIO conformation.

The contact interaction map for the very potent compound **19** identifies residues that may be exposed selectively in the IOP conformation (Figure 8). In our docking study, the interactions surrounding inhibitors for the different conformations of GLUT1 are dissimilar. The obvious distinction among the different GLUT1 conformations is the rotation of Trp388 around the binding site. In the IOP conformation, Trp388 is close to the ligands and more likely to interact and form H-bonds. In addition, Phe379 and Trp412 in the IOP conformation are readily accessible for ligand interactions. In contrast, Phe379, Trp388, and Trp412 in the OOP and POO conformations are away from the binding site and, thus, unable to participate in ligand binding interactions. Trp388 in the PIO conformation almost starts to drift away from the binding site whereas Phe379 and Trp412 are far from the binding site. In our docking study, the contact interaction map in the IOP conformation showed that complex of transmembrane helixes (TMs) TM5, TM7, TM10, and TM11 surround the ligand while in the IPO conformation the ligand is mostly surrounded by TM7, TM10, and TM11. In contrast, the ligand is bounded by TM1, TM5, and TM7 in the OOP conformation and TM5, TM7, TM8, and TM10 in the OOC conformation (Figure 8).

Moreover, the contact interaction of the IOP conformation reveals hydrophobic and polar residues in the binding site sharing hydrogen bonds and π–π stacking with the inhibitor. Furthermore, the TM10 is exposed in the active site and can interact with inhibitors for binding in the IOP conformation. This explains why inhibitors interact with Phe379, Trp388, and Trp412 mainly in IOP conformation, and this provides a rational clarification that the IOP conformation is favorable for productive binding interactions.

**Figure 9:**
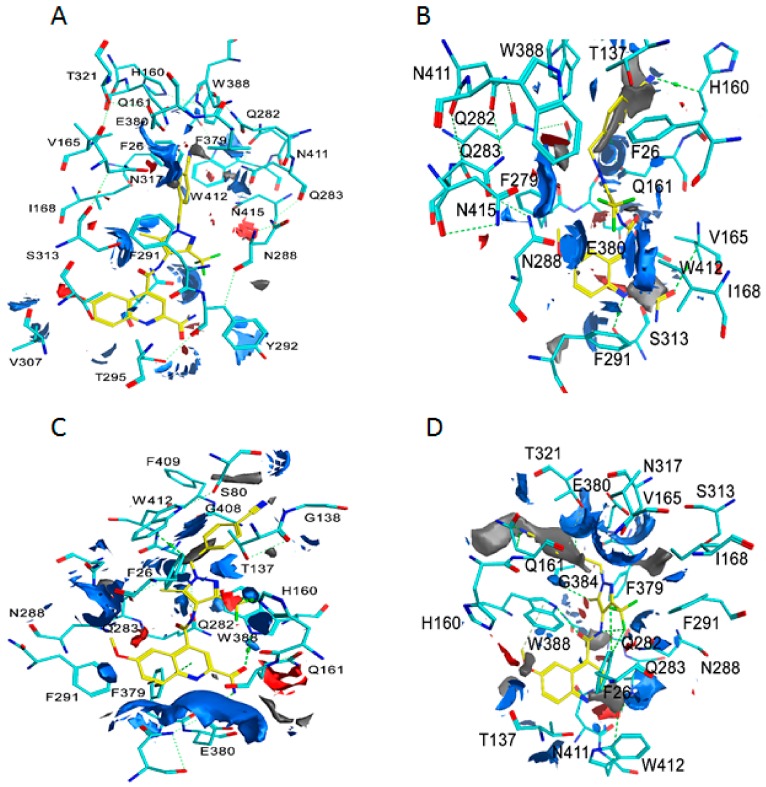
Electrostatic map between 19 and GLUT1 in different conformations: The H-bond interactions are shown as green dotted blue. (**A**) The OOP conformation. (**B**) The POO conformation. (**C**) The IOP conformation. (**D**) The PIO conformation.

The electrostatic map of GLUT1 reveals that the most prominent difference between the various protein conformations lies in Trp388; the nitrogen atom of indole ring of Trp388 is located toward the binding site and functions as an H-bond donor to an inhibitor in the IOP conformation. The carboxyl group of residue Glu380 serves as H-bond donor to the amide functional group of compound 19 (Figure 9C) whereas these two H-bonds are absent in other conformations. The Trp388 in the PIO conformation mostly starts to flip out the binding site, and this makes Trp388 less likely to form H-bond with the ligands. However, the Trp388 in the OOP and POO conformations is distant and unlikely to interact with inhibitors. This suggests that the indole ring of Trp388 is important for IOP selective binding. In addition, in the IOP conformation, Phe291, Phe379, Glu380, and Trp412 play important roles in ligand binding by forming π–π stacking and favorable H-bond interactions with inhibitors. We conclude that inhibitors interact with Trp388 and Glu380 via H-bonds, and with Phe291, Phe379, and Trp412 by π–π stacking in the IOP conformation (Figure 7, Figure 8 and Figure 9). 

## 3. Materials and Methods

Only wild type (WT)-human GLUT1 (hGLUT1) inward-open (IOP) conformation has been resolved in X-ray crystal structures (PDB: 4PYP, 5EQG, 5EQH, and 5EQI) [5,32]. For other unavailable human model proteins, we used the following homology modeling method to build various types of conformations. Several atoms structures of GLUTs have been resolved with different conformational states and/or different subtypes: Bacterial GLUTs homologous such as the d-xylose:H1 symporter from *Escherichia coli* (XylE) in partial outward occluded (POO) conformation (PDB: 4GBY, 4GBZ, and 4GC0) [43]; and partial inward occluded (PIO) conformations (PDB: 4JA3) [44]; and the GLUT1 homologs of the lactose permease of *Escherichia coli* (LacY) [51], and the glucose:H1 symporter from *Staphylococcus epidermidis* (GlcP) [52]. The hGLUT3 outward-open (OOP) and outward-occluded (OOC) conformations were identified with X-ray structures with PDB IDs of 4ZWC and 4ZW9, respectively [4]. The essential amino acids for the interacting with glucose are invariant between XylE and GLUT1 and listed in Figure S1 [43]. Therefore, the hGLUT3 and the XylE are GLUT1 homologs and they can be an appropriate template to build respective conformational states of hGLUT1. 

### 3.1. Homology Modeling and Preparation of Model Proteins

We used five structures of several conformations for GLUT1 for docking studies. For the IOP conformation, we downloaded and utilized the X-ray crystal structures of hGLUT1 (PDB ID:5EQG), which bound to phenylalanine amide compound (5RE) [32] from the RCSB Protein Data Bank at http://www.rcsb.org/pdb/. No hGLUT1 X-ray structures have been reported yet for the OOP, POO, OOC, and PIO conformations. Thus, homology model techniques were employed. Sequence alignments used for building the homology model are listed in Figure S1. Strong homology was identified between hGLUT3 and hGLUT1, and XylE and hGLUT1 by ProBiS-CHARMMing [53]. Structural alignment between these three proteins using the Clustalo program [54] (Version: 1.2.4) further confirmed the structural homology between them. The structural alignment is illustrated in Figure 1. The following protein templates were used to build different conformational states: hGLUT3 (PDB ID: 4ZW9, and 4ZWC) [4] for the OOC and OOP conformations; XylE (PDB ID: 4GBZ, and 4JA3) [43,44] for the POO and PIO conformations. The ligand (β-NG) was adopted from (PDB ID: 4PYP) [5] as a ligand for 4JA3 homology model. There are four regions of missing residues in the crystal structure of 4JA3: Region 1 (Lys265–Val275), region 2 (Phe304–Ala309), region 3 (Ile398–Lys406), and region 4 (Trp434–Phe439). The missing residues were constructed by using loop modeler on MOE [55] within root mean square deviation (RMSD) limit = 0.5 Å. The template for region 1 and region 3 was selected from (PDB ID: 3TZY.A) [56] and (PDB ID: 3RE4.A) [57], respectively. The templates for region 2 and region 4 were built by *de novo* method. The rebuilt residues that were missing and its surrounding residues underwent energy minimization to minimize steric repulsion. In addition, all these protein structures were subjected to automated structure preparation to fix issues found in crystallographic structure such as replacing missing protein sections, optimization of the hydrogen bonding network, and allowing protonation be assigned to charged residues and allowing the flipping side chains of Asn, Gln, His in MOE to maximize H-bond interactions. Also, they were subjected to energy minimization using the Amber14:EHT [58] force field in MOE, followed by protein preparation using the Protein Preparation Wizard in the Schrödinger software to allow the side-chain of residues of Asn and Gln to move to make the most favorable H-bond interactions. Then, they were subjected to energy minimization with protein backbone by using the OPLS3 force field in the MacroModel module in the Maestro 11.2 [59]. The stereochemical quality of homology modeling was evaluated by the Ramachandran’s plot assessment. The Ramachandran’s plot assessment was measured by MOE [54] and Procheck [60] and were listed in Figure S2 in the Supplementary Materials.

### 3.2. Ligands Sources, Prepration, and Docking

The small molecules (Figure 2, Figure 3, Figure 4 and Figure 5) were built using MOE build panel and subjected to energy minimization using the MMFF94x force field in MOE using the MMFF force field partial charges. In addition, they were minimized by using the OPLS3 force field of the MacroModel program using the OPLS3 force field partial charges. All ligands were subject to pKa calculations using the Epik program in the Schrödinger software. The Epik calculations [59] showed that the pKa of all nitrogen atoms in these ligands are less than 7, suggesting that under the pH = 7 condition, all nitrogen shall remain deprotonated, i.e., neutral. The characteristic pKa values for molecules were listed in Figure 4. A database of 260,071 ligands was taken from the National Cancer Institute database [61] and further filtered by the logP (logP < 5), and molecular weight (MW < 500) set by the Lipinski’s rule of five (RO5) [62]. To expedite the docking and to define enrich factors, we randomly selected 508 drug-like molecules from this database after the filtration of RO5. These 508 molecules were minimized using the OPLS3 force field and the OPLS3 partial charges using the MacroModel program. 

Commonly used software for ligand-protein docking include the Surflex docking module in Sybyl [63,64], and the Schrödinger Glide Dock program [65,66]. In this study for ligand docking to GLUT1, we used Glide Dock [59] in the Maestro 11.2 with the different conformations of GLUT1 (5EQG, HM_4ZW9, HM_4ZWC, HM_4GBZ, and HM_4JA3) as target proteins. First, crystallographic water and 1-Oleoyl-R-glycerol (OLC) molecules were deleted from 4ZW9, 4ZWC, and 4GBZ. Hydrogen atoms were added to both the protein and the ligand. Then, five grid files for 5EQG, 4ZW9, 4ZWC, 4GBZ, and 4JA3 were created by the Glide Grid Generation panel [59] with the bound ligands as the centroid of the protein binding pocket. The site of the bound ligands in the crystal structure 5EQG was defined as the centroid; and for the homology models, all four homology models were structurally aligned to 5EQG and, thus, the 5EQG-bound ligand was adopted as bound ligand for those four homology models and used as a centroid to define binding pockets for those four homology models. Then, all GLUT1 inhibitors were docked with the precision and ligand sampling were set to extra-precision (XP) method to generate each of the five grid files. All other parameters were used as defaults. In addition, 508 drug-like molecules were also docked to the different conformations of GLUT1 (5EQG, 4ZW9, 4ZWC, 4GBZ, and 4JA3). The binding affinity of the various conformation of GLUT1/ligand complexes was evaluated by the Glide scores. The protein/ligand interactions and contact interaction were created by using the Maestro 11.2. The electrostatic map was generated by using the MOE. The frequency of residues interacting with ligands illustrated in Figure 7 was made using Excel.

## 4. Conclusions

In summary, protein–ligand dockings on different GLUT1 conformations suggest three significant outcomes: First, the docking scores suggested that the IOP conformation would be preferred for ligand binding. Besides, the MAE and RMSE for each conformation in comparison to experimental observations IC_50_ of ligands also confirm this conclusion. Second, enrichment factor (EF) calculation from docking studies further confirms that the Glide dock program is able to distinguish the real inhibitors from drug-like molecules with best results in the IOP conformation. Third, residues Trp388, Glu380, Phe379, and Trp412 are important for the IOP conformation selective binding. Taken together, all these results support the conclusion that the IOP conformation is the most recognized conformation for ligand interaction and, thus, should be used for future molecule design targeting GLUT1. 

## Figures and Tables

**Table 1:** Glide scores (Kcal/mol) of GLUT1 inhibitors against different conformation models of GLUT1: 5EQG (IOP); HM_4GBZ (POO); HM_4ZWC (OOP); and HM_4JA3 (PIO).

Compd.	IC_50_ (uM) ^a^	∆G_exp_^b^	5EQG	ΔΔG(5EQG)	HM_4GBZ	HM_4ZWC	HM_4JA3
**1**	0.11	−9.49	−9.39	−0.10	NA ^c^	−5.84	NA ^c^
**2**	0.267	−8.97	−10.61	1.64	−11.63	−11.11	−10.53
**3**	0.14	−9.35	−11.54	2.19	−9.46	−10.26	−10.84
**4**	0.11	−9.49	−9.53	0.04	−9.05	−8.88	−6.64
**5**	0.006	−11.22	−9.39	−1.83	−7.22	−7.71	−7.96
**6**	0.004	−11.46	−9.24	−2.22	−7.45	−9.11	−8.23
**7**	0.003	−11.63	−9.45	−2.18	−9.77	−10.23	−8.49
**8**	0.008	−11.05	−9.68	−1.37	−9.61	−10.16	−8.35
**9**	0.087	−9.63	−9.43	−0.20	−7.39	−8.75	−8.73
**10**	0.002	−11.87	−9.24	−2.63	−8.83	−8.10	−8.17
**11**	0.004	−11.46	−10.62	−0.84	−10.34	−9.01	−10.23
**12**	0.032	−10.22	−10.30	0.08	−9.93	−8.74	−10.12
**13**	0.005	−11.32	−10.96	−0.36	−9.36	−9.18	−9.68
**14**	0.026	−10.35	−10.38	0.03	−8.99	−9.10	−9.42
**15**	0.006	−11.22	−9.49	−1.73	−9.47	−9.17	−9.49
**16**	0.003	−11.63	−11.47	−0.16	−10.22	−9.26	−9.34
**17**	0.005	−11.32	−9.34	−1.98	−10.36	−9.51	−10.99
**18**	0.007	−11.12	−10.29	−0.83	−10.88	−9.02	−9.32
**19**	0.0003	−12.99	−9.61	−3.38	−10.91	−9.77	−10.78
**20**	0.002	−11.87	−9.70	−2.17	−9.71	−9.51	−10.44
**21**	0.008	−11.05	−8.92	−2.13	−10.95	−10.03	−9.78
**22**	0.024	−10.39	−9.52	−0.87	−9.12	−9.72	−11.13
**23**	0.005	−11.32	−10.32	−1.00	−10.24	−9.84	−10.00
**24**	0.003	−11.63	−10.34	−1.29	−10.94	−9.96	−9.68
**25**	0.0005	−12.69	−11.37	−1.32	−10.46	−9.04	−10.77
**26**	0.002	−11.87	−7.42	−4.45	−10.11	−9.39	−11.14
**27**	0.014	−10.71	−10.82	0.11	−10.20	−10.56	−9.65
**28**	0.01	−10.91	−10.49	−0.42	−9.20	−9.42	−9.88
**29**	0.045	−10.02	−9.33	−0.69	−10.25	−9.61	−9.26
**30**	0.004	−11.46	−9.48	−1.98	−10.02	−10.45	−10.28
**31**	0.76	−8.35	−9.12	0.77	−4.47	−9.53	−8.30
**32**	0.007	−11.12	−9.25	−1.87	−9.83	−9.23	−10.18
**33**	0.074	−9.73	−9.54	−0.19	−5.26	−10.13	−10.32
**34**	0.92	−8.23	−10.62	2.39	−9.99	−9.42	−10.60
**35**	0.34	−8.82	−11.77	2.95	−12.23	−11.43	−12.81
**36**	0.0009	−12.34	−9.02	−3.32	−9.99	−9.18	−9.65
**37**	0.003	−11.63	−9.70	−1.93	−9.75	−9.53	−8.38
**38**	0.007	−11.12	−8.89	−2.23	−9.81	−8.92	−9.61
**39**	0.007	−11.12	−10.36	−0.76	−9.83	−8.64	−9.40
**40**	0.003	−11.63	−9.45	−2.18	−9.84	−10.10	−10.28
**41**	0.007	−11.12	−10.09	−1.03	−8.71	−10.13	−9.73
**42**	0.009	−10.98	−11.28	0.30	−10.07	−10.32	−10.81
**43**	0.005	−11.32	−10.58	−0.74	−9.91	−10.01	−9.84
**44**	0.0009	−12.34	−9.59	−2.75	−9.20	−9.38	−10.89

Note: ^a^ The IC_50_ were obtained from Reference 32, and 36. ^b^ ΔG_exp_ = *RTln(IC_50_* × 10^−6^)/1000 for IC_50_s with µM unit and ΔG_exp_ in Kcal/mol unit, whereas R: Universal gas constant (1.987 cal/mol.K), T: Temperature in Kelvin (298.15 K). ^c^ NA: no docked pose was available.

**Table 2:** The average of the mean errors between experimental free energy (ΔG_exp_) and predicted free energy for different models: 5EQG (IOP); HM_4GBZ (POO); HM_4ZWC (OOP); and HM_4JA3 (PIO).

	5EQG (IOP)	4GBZ (POO)	4ZWC (OOP)	4JA3 (PIO)
∆∆G	−0.97	−1.37	−1.44	−1.16
MAE	1.45	1.75	1.82	1.66
RMSE	1.79	2.07	2.05	1.92

Note: MAE: Mean absolute errors; RMSE: Root mean square errors.

**Table 3:** The enrichment factors of docking scores for different conformations: The ideal enrichment factor (EF) in this case equals 13.44.

Model	EF (1%)	EF (5%)	EF (10%)
5EQG	10.45	8.51	7.76
4GBZ	4.24	3.30	4.63
4ZWC	8.35	8.05	7.06
4JA3	6.37	8.02	6.72

## Supplementary Materials

The supplementary materials are available online.
