# Supplementary material for: Conformational Studies of Glucose Transporter 1 (GLUT1) as an Anticancer Drug Target
Source: Molecules. 2019 Jun 7;24(11):2159. doi: 10.3390/molecules24112159 (PMC6600248; doi:10.3390/molecules24112159)
Supplement: Supplementary file 1 [file molecules-24-02159-s001.pdf]

Supporting Information

Conformational Studies of Glucose Transporter 1 (GLUT1) As Anticancer Drug Targets

Suliman Almahmoud[1], Xiaofang Wang[1], Jonathan L. Vennerstrom[1],Haizhen A. Zhong[2]*

[1] College of Pharmacy, Universty of Nebraska Medical Center, Omaha, Nebraska 68198, United States
[2] Department of Chemistry, University of Nebraska at Omaha, Omaha, Nebraska, United States
* Corresponding author: H.A. Zhong, hzhong@unomaha.edu; Tel.: (1) (402) 554-3145

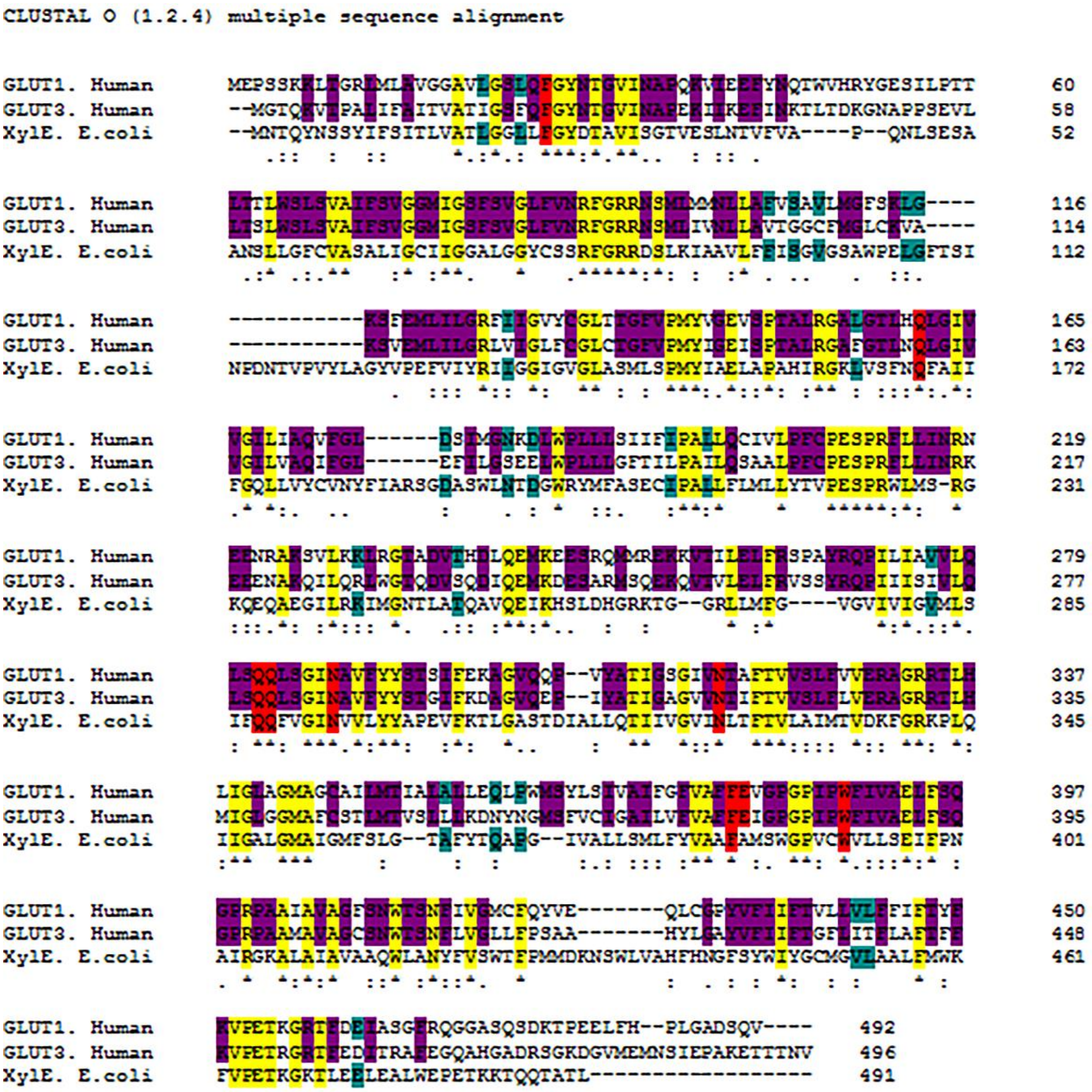

Figure S1. Primary sequence alignment of GLUT1, GLUT3, and XylE**:** Invariant and highly conserved residues between GLUT1, GLUT3, and XylE are highlighted yellow; conserved residues between GLUT1 and GLUT3 are highlighted violet; conserved residues between GLUT1 and XylE are highlighted teal. The amino acid residues that are bonded to D-glucose are highlighted red. The human GLUT3 were used to build the homology modeling of OOP and OOC conformations, while bacterial XylE were used to build the homology modeling of POO and PIO conformations.

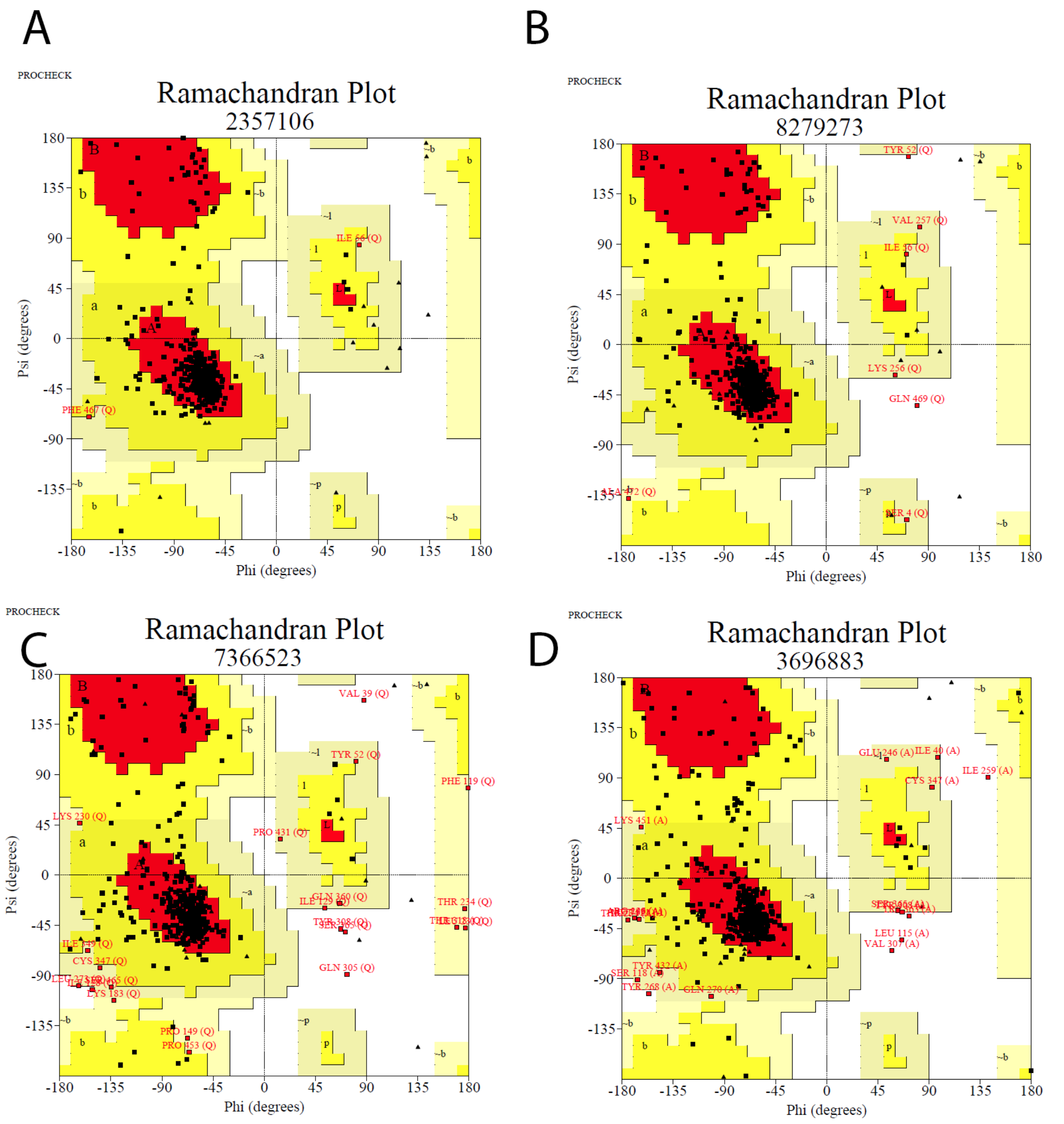

Figure S2. The Ramachandran plots for the four homology models and the number of residues in the disallowed regions**:** (**A**) The OOP conformation (4ZWC model: zero residues in disallowed regions); (**B**) the OOC conformation (4ZW9 model: Only 2 out of 404 residues (0.5%) in the disallowed regions: Tyr52 and Gln469); (**C**) the POO conformation (4GBZ model: 5 out of 398 residues (1.3%) in the disallowed regions: Val39, Ile128, Gln305, Tyr308, and Ser365); (**D**) The PIO conformation (4JA3 model: 6 out of 383 residues (1.6%) in the disallowed regions: Leu115, Ile259, Tyr268, Val307, Tyr308, and Trp363)

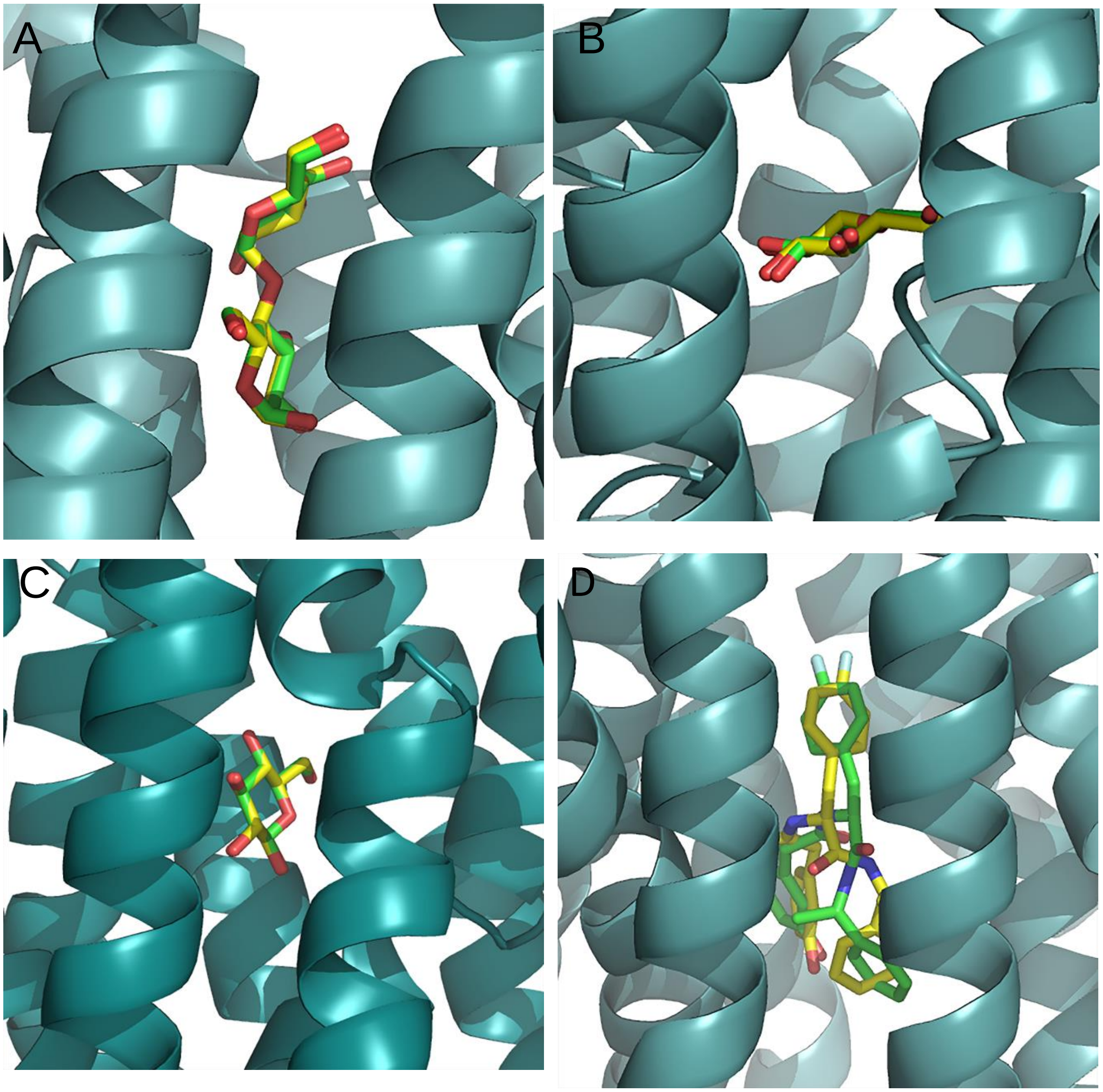

Figure S3. The superposition of the Glide generated docked poses and the native conformation of ligand in the crystal structure**: A)** MAL in 4ZWC, **B)** BGC in 4GBZ, **C)** BGC in 4ZW9, **D)** 5RE in 5EQG. Color code: Yellow, the lingand in the crystal structure; green, the lingand fro Glide generated poses. The RMSD between two poses for MAL in 4ZWC, BGC in 4GBZ, BGC in 4ZW9, and 5RE in 5EQG is 0.14, 0.61, 0.06, and 1.62 Å, respectively.

Table S1. Glide scores of 508 drug-like molecules obtained from an NCI database against the GLUT1 inward-open conformation**.**

NCS Title	Glide Score	NCS Title	Glide Score	NCS Title	Glide Score	NCS Title	Glide Score
**451**	-3.873	**76604**	-4.336	**194897**	-5.433	**611435**	-8.255
**1282**	-6.413	**76808**	-7.643	**195326**	-6.453	**611913**	-10.207
**1439**	-4.227	**76925**	-7.133	**201533**	-7.244	**613729**	-8.14
**1642**	-5.464	**77128**	-5.198	**202825**	-5.252	**615396**	-7.67
**1971**	-7.39	**77739**	-7.642	**203330**	-5.558	**615397**	-8.353
**3248**	-2.655	**79245**	-8.839	**204342**	-6.704	**617827**	-4.404
**3581**	-9.254	**79575**	-6.506	**204799**	-9.066	**618430**	-8.198
**3604**	-6.474	**81217**	-5.357	**205707**	-7.787	**618508**	-9.099
**3609**	-3.837	**81336**	-4.566	**207116**	-5.409	**619691**	-9.732
**4092**	-2.982	**81767**	-6.761	**208702**	-6.595	**620117**	-6.249
**4863**	-6.921	**82222**	-8.339	**208881**	-3.392	**620148**	-9.922
**5037**	-3.623	**86416**	-7.947	**211578**	-8.428	**620280**	-8.225
**5317**	-5.354	**92983**	-4.829	**212090**	-7.858	**622374**	-7.742
**5431**	-3.497	**93303**	-7.837	**212139**	-6.878	**622562**	-7.489
**6846**	-8.354	**93438**	-6.207	**213630**	-8.539	**623439**	-5.991
**7796**	-9.607	**93714**	-5.258	**215613**	-6.592	**623762**	-7.567
**8218**	-5.058	**93929**	-5.088	**216752**	-7.675	**623898**	-7.348
**8588**	-8.165	**95384**	-5.466	**217039**	-3.418	**626384**	-8.544
**9430**	-4.876	**95570**	-6.671	**220094**	-5.586	**626940**	-7.37
**9489**	-4.749	**95791**	-6.404	**220240**	-4.614	**630876**	-7.396
**10080**	-5.552	**96341**	-8.957	**222625**	-5.57	**631647**	-4.989
**10752**	-8.41	**96992**	-7.229	**222790**	-4.028	**633214**	-5.674
**11429**	-6.697	**97507**	-2.741	**226089**	-5.275	**634581**	-4.59
**13036**	-8.21	**98375**	-5.546	**229350**	-4.7	**634627**	-7.156
**13141**	-5.575	**100941**	-6.797	**229647**	-5.245	**636507**	-9.912
**13961**	-4.936	**102391**	-7.71	**231557**	-4.931	**636881**	-7.444
**13997**	-5.003	**102541**	-4.347	**236249**	-6.968	**637401**	-7.348
**14001**	-6.007	**103747**	-4.501	**241598**	-7.575	**641124**	-7.748
**15330**	-5.14	**105534**	-7.356	**243021**	-7.968	**641190**	-8.224
**15440**	-6.291	**106060**	-8.192	**244299**	-2.431	**641860**	-7.262
**16766**	-10.046	**106310**	-6.252	**245089**	-4.55	**644223**	-9.246
**16886**	-4.84	**107092**	-6.443	**245146**	-7.174	**645810**	-7.249
**17233**	-3.73	**107109**	-4.602	**248531**	-9.012	**645901**	-8.09
**17461**	-5.956	**107160**	-8.707	**249213**	-4.935	**646436**	-7.012
**17682**	-4.041	**108078**	-6.405	**250686**	-10.463	**646652**	-7.907
**17823**	-4.747	**108395**	-7.277	**251159**	-6.049	**647155**	-8.86
**19082**	-4.604	**108611**	-7.299	**260419**	-6.255	**647621**	-7.581
**19676**	-5.691	**109297**	-6.869	**264255**	-9.231	**648213**	-7.361
**19929**	-5.156	**110619**	-6.644	**264430**	-4.989	**648842**	-9.067

**20654**	-4.884	**110651**	-9.085	**265436**	-7.42	**649595**	-5.748
**21347**	-7.813	**110770**	-7.359	**267447**	-6.956	**650736**	-7.849
**21468**	-4.848	**111712**	-8.327	**268719**	-4.982	**652037**	-7.732
**21770**	-6.202	**111724**	-5.616	**270703**	-6.266	**652117**	-7.327
**21941**	-5.01	**112430**	-6.638	**271266**	-5.934	**652910**	-7.9
**22940**	-8.785	**113532**	-5.241	**276740**	-8.007	**652938**	-9.281
**23086**	-6.681	**114477**	-5.964	**278073**	-5.953	**653492**	-5.888
**23458**	-7.639	**114640**	-6.082	**283482**	-7.285	**653956**	-7.227
**23538**	-4.636	**115165**	-8.206	**284220**	-5.503	**654111**	-4.627
**23700**	-4.658	**115221**	-4.203	**284670**	-6.757	**655335**	-7.327
**24991**	-4.78	**115615**	-6.806	**286671**	-7.312	**657952**	-7.786
**25039**	-6.275	**115932**	-6.911	**286710**	-7.136	**657983**	-7.574
**26460**	-3.62	**116803**	-3.909	**287491**	-8.624	**658812**	-8.309
**26836**	-7.015	**117260**	-6.192	**287993**	-4.439	**659307**	-8.055
**26960**	-5.55	**117379**	-4.896	**289365**	-6.377	**660842**	-6.791
**27907**	-8.106	**118712**	-7.416	**290094**	-10.025	**661173**	-6.658
**28401**	-7.327	**119498**	-5.081	**290494**	-8.608	**661954**	-7.626
**29324**	-7.836	**119542**	-5.498	**291066**	-5.726	**662770**	-5.732
**29874**	-5.403	**120511**	-5.572	**291572**	-8.339	**664213**	-9.352
**30159**	-6.362	**122582**	-7.64	**293788**	-4.928	**665489**	-8.854
**30215**	-4.025	**122619**	-2.924	**294379**	-4.106	**666377**	-7.761
**32052**	-8.323	**123133**	-3.17	**294992**	-8.329	**666861**	-7.5
**32568**	-8.177	**123278**	-4.729	**297929**	-6.375	**669315**	-9.568
**33614**	-7.738	**123820**	-6.011	**299964**	-9.223	**671104**	-9.276
**33632**	-4.201	**124206**	-8.455	**300236**	-4.443	**671327**	-7.92
**33634**	-5.652	**124616**	-5.677	**300908**	-5.744	**671802**	-8.209
**33698**	-4.559	**125017**	-8.328	**301466**	-2.996	**672556**	-6.313
**35498**	-6.365	**125376**	-5.984	**305213**	-7.513	**675799**	-8.283
**36819**	-10.133	**125563**	-8.905	**306131**	-7.985	**676446**	-8.728
**37853**	-5.397	**125618**	-7.725	**306778**	-5.384	**677448**	-9.409
**41519**	-6.725	**126880**	-7.123	**308800**	-7.223	**677792**	-9.071
**41633**	-6.033	**128910**	-4.525	**312029**	-6.553	**679560**	-5.866
**41814**	-6.735	**131082**	-6.077	**312610**	-7.416	**679577**	-4.884
**42019**	-5.812	**131164**	-5.144	**319030**	-7.34	**683715**	-4.869
**42597**	-6.194	**131287**	-5.344	**332690**	-6.458	**686789**	-5.412
**42740**	-5.45	**132945**	-6.831	**338058**	-7.571	**687820**	-8.18
**43063**	-6.254	**134178**	-8.908	**338186**	-4.371	**687853**	-8.375
**43639**	-4.168	**135021**	-7.466	**338531**	-8.441	**688729**	-7.174
**44348**	-4.783	**135066**	-8.671	**338574**	-5.474	**689594**	-5.103
**45766**	-4.564	**135311**	-5.919	**338604**	-5.329	**690572**	-5.547
**47429**	-5.592	**137412**	-5.275	**338623**	-6.875	**691569**	-7.634
**48174**	-5.181	**137505**	-4.964	**338627**	-6.405	**691849**	-8.709
**49499**	-8.107	**139207**	-8.346	**344016**	-8.089	**692611**	-5.82

**49745**	-6.402	**140053**	-8.907	**345395**	-7.311	**693633**	-6.141
**51876**	-6.03	**140382**	-5.726	**346098**	-6.434	**696124**	-9.281
**52216**	-3.921	**141876**	-4.713	**352267**	-8.091	**697931**	-9.113
**52479**	-6.426	**142200**	-7.269	**355186**	-10.336	**698074**	-7.522
**53077**	-5.706	**142555**	-8.379	**356130**	-6.998	**698559**	-5.309
**53284**	-5.223	**143308**	-7.863	**356486**	-6.496	**699123**	-6.562
**53466**	-6.044	**143989**	-5.357	**357566**	-6.611	**703459**	-7.339
**53806**	-5.63	**145881**	-5.164	**362252**	-4.929	**703889**	-8.367
**53880**	-5.454	**147640**	-6.422	**363847**	-2.987	**704117**	-4.31
**54427**	-9.253	**148291**	-8.393	**364027**	-6.257	**708438**	-5.699
**55495**	-5.542	**149692**	-6.317	**364095**	-4.929	**708967**	-6.923
**56629**	-6.838	**150016**	-4.771	**364720**	-7.067	**709508**	-6.795
**57381**	-6.074	**150158**	-6.961	**367101**	-7.838	**714085**	-8.228
**57543**	-3.072	**151034**	-6.601	**368255**	-8.255	**714378**	-7.249
**58322**	-8.723	**151087**	-7.556	**372636**	-8.08	**715654**	-8.604
**58812**	-3.608	**151259**	-5.509	**372663**	-3.917	**716023**	-4.277
**58894**	-6.522	**151736**	-7.105	**372778**	-8.555	**716062**	-9.053
**59022**	-4.885	**152426**	-7.784	**374153**	-2.617	**719712**	-7.447
**60443**	-6.719	**152647**	-7.731	**382808**	-8.786	**720995**	-8.626
**60806**	-6.769	**154776**	-6.229	**382958**	-10.624	**724571**	-6.661
**62525**	-7.98	**156773**	-8.563	**402506**	-5.26	**724869**	-6.906
**62992**	-4.386	**158180**	-6.024	**403474**	-6.841	**725149**	-9.189
**63031**	-4.387	**160062**	-7.281	**404215**	-8.188	**726399**	-8.402
**63486**	-7.476	**160173**	-4.926	**404221**	-4.446	**726406**	-9.176
**65372**	-9.392	**162174**	-4.675	**404935**	-4.211	**726719**	-6.941
**65988**	-4.171	**162275**	-5.077	**406312**	-4.306	**727512**	-9.435
**66471**	-9.569	**162289**	-4.709	**406401**	-5.075	**727978**	-10.419
**66524**	-4.754	**163853**	-4.545	**406476**	-3.607	**728240**	-10.543
**66596**	-5.113	**164081**	-8.689	**408503**	-7.797	**729188**	-7.389
**67195**	-3.594	**167493**	-4.489	**408859**	-5.866	**730571**	-7.933
**67458**	-5.462	**167911**	-4.692	**409231**	-6.293	**730774**	-8.501
**67470**	-7.888	**169683**	-7.57	**409518**	-6.651	**731659**	-8.504
**67854**	-6.018	**169879**	-5.098	**508388**	-3.955	**732842**	-8.313
**68482**	-5.439	**171178**	-4.717	**511710**	-4.255	**733410**	-7.218
**70306**	-5.684	**171545**	-9.257	**512733**	-9.2	**734148**	-7.154
**70656**	-4.039	**171619**	-4.324	**515544**	-6.457	**740535**	-7.664
**70958**	-6.681	**172291**	-6.217	**515547**	-7.301	**740617**	-8.264
**71385**	-2.858	**172636**	-7.434	**516423**	-6.185	**743490**	-7.926
**72069**	-4.947	**173085**	-7.405	**522167**	-5.008	**744263**	-7.799
**72797**	-8.747	**173346**	-4.103	**523371**	-4.576	**744479**	-4.436
**73945**	-3.568	**173353**	-4.604	**526972**	-5.464	**745068**	-7.698
**74632**	-4.639	**173742**	-3.931	**606256**	-9.268	**746579**	-7.658
**75421**	-4.902	**179467**	-11.576	**608686**	-7.499	**748262**	-8.274

**75952**	-11.169	**179618**	-4.76	**608833**	-6.896	**748543**	-7.607
**76503**	-6.905	**185315**	-6.97	**610980**	-7.464	**748706**	-8.022

Table S2. Residues interaction with different GLUT1 inhibitors**:** 5EQG: Inward-open conformation; HM_4GBZ: Outward-partially occluded conformation; HM_4ZWC: Outward-open conformation; HM_4JA3: Inward-partially occluded conformation.

[a]NA: No interaction was found between the ligand and the GLUT1.

Compd	5EQG	HM_4GBZ	HM_4ZWC	HM_4JA3
**1**	Thr137, His160, Gln282, Gln283, Trp388, Gly384	NA[a]	Asn34, Gln283, Asn288, Asn415	NA[a]
**2**	His160, Trp388	Gly157, Gln161	Asn317, Phe379, Glu380	Asn288, Asn317, Asn411
**3**	His160, Trp388	Phe291, Asn317	Gln161	Trp388
**4**	Phe26, His160, Phe379, Trp388	Phe26, Phe291	NA[a]	Phe26, Trp388
**5**	Phe26, His160, Phe389	Phe26	NA[a]	Trp412
**6**	His160, Trp388	Phe291	NA[a]	Trp388, Trp412
**7**	His160, Phe379	Phe291	Gln161, Phe379	Trp388
**8**	His160, Phe379	Phe291	Gln161, Phe379	Trp388
**9**	Asn288, Trp388	Phe291	NA[a]	Phe291, Trp388, Trp412
**10**	His160, Phe389	Phe291	Asn34, Asn288	Trp388
**11**	Phe26, Phe291, Phe379, Glu380, Trp388, Trp412	Ser313	Gln37, Gln282, Gln283, Asn288	Asn288, Asn317, Thr321, Trp388
**12**	Glu380, Trp388, Trp412	Ser313	Gln37, Gln161	Tyr292, Trp388
**13**	Phe26, Phe291, Phe379, Glu380, Trp388, Trp412	Ser313	Gln161, Gln172	Tyr292, Trp388
**14**	Phe26, Phe291, Phe379, Glu380, Trp388, Trp412	Ser313	Gln161, Gln172	Asn288, Asn317, Trp388
**15**	Phe379, Glu380, Trp388, Trp412	Ser313	Gln37	Trp388, Trp412
**16**	Phe26, Phe291, Phe379, Glu380, Trp388, Trp412	Ser313	Asn34, Gln37, Gln161, Tyr292	Asn288, Trp388
**17**	Phe26, Phe379, Glu380, Trp388, Trp412	Ser313	Tyr292, Val418	Trp388, Asn415
**18**	Phe26, Phe291, Phe379, Glu380, Trp388, Trp412	Ser313	Gln172, Asn288	Tyr292, Trp388
**19**	Phe26, Phe379, Glu380, Trp388, Trp412	Phe291, Ser313	Gln37	Tyr292, Trp388, Asn415
**20**	Phe379, Glu380, Trp388, Trp412	Ser313	Gln37, Arg126	Trp388, Asn415
**21**	Phe26, His160, Trp388, Phe389	Phe291	Asn34, Asn317	Asn288, Asn317, Trp388
**22**	Phe26, Trp388	Ser313	Asn34, Asn317	Tyr292, Trp388, Asn415
**23**	Phe26, Phe291, Phe379, Glu380, Trp388, Trp412	Ser313	Asn317	Tyr292, Trp388
**24**	Glu380, Trp388	Phe26, Ser313	Gln37, Asn317	Asn288, Trp388
**25**	Phe26, Phe291, Phe379, Glu380, Trp388, Trp412	Ile287, Phe291	Gln37, Gln283	Asn317, Trp388

**26**	Phe26, Phe379, Glu380, Trp388, Trp412	NA[a]	Gln172, Asn288	Asn317, Trp388, Asn415
**27**	Phe26, Phe291, Phe379, Glu380, Trp388, Trp412	Thr310, Ser313	Asn317	Asn288, Trp388
**28**	Phe26, Phe291, Phe379, Glu380, Trp388, Trp412	Thr310, Ser313	Asn317	Tyr292, Trp388
**29**	Phe26, Phe379, Glu380, Trp388, Trp412	Ser313	Gln37, Gln161, Asn317, Phe379	NA[a]
**30**	His160, Phe389	Ser313	Asn34	Asn317
**31**	Phe26, His160	Phe291	Gln161, Asn288, Phe379	Trp388, Trp412
**32**	Phe26, His160, Trp388	Phe291	Gln37, Gln161, Asn288, Phe379	Asn288, Asn317, Trp388
**33**	His160, Trp388	Phe291	Gln37, Tyr292, Asn317	Asn317, Trp388
**34**	Phe26, His160, Trp388, Phe389	Phe26	NA[a]	Asn317, Trp388
**35**	Phe379, Glu380, Trp388, Trp412	Ser313	Asn34, Gln37, Asn317	Asn288, Asn317, Trp388
**36**	Phe379, Glu380, Trp388	Ser313	Gln37	Asn288, Asn317, Trp388
**37**	Phe26, Phe291, Phe379, Glu380, Trp388, Trp412	Phe291, Ser313	Gln37, Gln161, Phe379	Tyr292, Trp388
**38**	Phe291, Phe379, Glu380, Trp388	Ser313	Asn34, Gln172	Tyr292, Phe379, Trp388
**39**	His160, Gln283, Asn288	Ser313	Gln283	Ala407
**40**	Ser80, Thr137, Asn288	NA[a]	Asn34, Asn288, Asn317, Asn415	Asn317, Glu380, Trp388
**41**	Phe26, Thr137, His160	Phe26, Asn288	Asn34, Gln172	Asn317, Trp388
**42**	Glu380, Trp388, Trp412	Ser313	Gln37	Asn288, Asn317, Thr321, Trp388
**43**	Phe379, Glu380, Trp388, Trp412	Ser313	Gln37	Asn288, Asn317, Thr321, Trp388
**44**	Phe26, Glu380, Trp388, Trp412	Ser313	Asn34	Asn317, Thr321, Trp388
